# The predictive and prognostic role of radiologically defined sarcopenia in head and neck cancer: a systematic review and multi-level meta-analysis

**DOI:** 10.1038/s41416-025-03049-7

**Published:** 2025-06-02

**Authors:** Hugo C. van Heusden, Maartje A. van Beers, Anouk W. M. A. Schaeffers, Emma Swartz, Justin E. Swartz, Remco de Bree

**Affiliations:** 1 Department of Head and Neck Surgical Oncology, Division Imaging and Oncology, University Medical Center Utrecht, Utrecht University, Utrecht, The Netherlands; 2 Department of Developmental Psychology, Utrecht University, Utrecht, The Netherlands; 3 Department of Otorhinolaryngology – Head and Neck Surgery, Diakonessenhuis, Utrecht, Utrecht, The Netherlands

**Keywords:** Head and neck cancer, Risk factors

## Abstract

Radiologically defined sarcopenia (RS), defined as a lack of skeletal muscle mass (SMM) measured on cross-sectional CT or MR imaging, is increasingly recognized as a significant prognostic determinant in head and neck cancer (HNC). A systematic literature search of Embase and Medline was performed to identify studies investigating the impact of pre-treatment sarcopenia on the prognosis of HNC patients. All available survival and other treatment-related outcomes were extracted and analyzed in a multi-level meta-analysis. Sixty-three studies comprising data from 14,804 patients were analyzed. The overall estimated log OR was 0.644 (95% CI = 0.505–0.783, *p* < 0.001), suggesting that patients with RS have a higher risk of worse outcomes. In 43 studies there was a significant effect of sarcopenia on survival, with a log OR of 0.808 (95% CI = 0.509–1.107, *p* < 0.001). In 15 studies RS was shown to be a risk factor for treatment-related complications (log OR = 0.669, 95% CI = 0.441–0.897, *p* < 0.001). We conclude that pre-treatment radiologically defined sarcopenia is a robust prognostic and predictive factor in HNC patients and is associated with worse survival and increased risk of treatment-related complications.

## Introduction

Head and neck cancer (HNC) ranks as the seventh most common cancer globally and its incidence is on the rise [1]. Treatment of HNC is multimodal involving surgery, radiotherapy, systemic therapy (i.e. chemotherapy or immunotherapy), or a combination thereof. Unfortunately, these treatment regimens are frequently associated with complications and subsequently decreased treatment tolerability [2]. Individuals with HNC are at an elevated risk of developing sarcopenia before start of treatment and during treatment due to increased susceptibility to malnutrition, associated comorbidities and treatment-related toxicities exacerbating these factors [3–6].

Sarcopenia is defined as the loss of skeletal muscle mass (SMM) and function and is associated with physical and functional impairment [7, 8]. Several methods exist to estimate whole-body SMM. One such method is to measure SMM using CT or MRI on a single cross-sectional slice. These measurements have been shown to be a strong predictor of whole-body SMM [9]. Radiologically defined sarcopenia is a deficit of SMM and often after adjustment for individual patient’s height presented as skeletal muscle index (SMI) [10]. It is an easily measured biomarker and is often retrospectively available. For this reason, most research omits the ‘function’ part of the definition and focuses on low SMM, which in itself has proven to be a significant predictive and prognostic factor. Henceforth, when referring to sarcopenia, we specifically denote radiologically defined sarcopenia for the sake of coherence in our study.

While recent systematic reviews and meta-analyses showed that sarcopenia is a negative prognostic factor for overall survival (OS), disease free survival (DFS), short-term treatment-related toxicity and postoperative complications, these studies often exhibit limitations in scope due to their focus on specific subdomains of HNC, measurement methods, or outcome factors. To provide a comprehensive overview of the available research, our review adopts a multi-level approach to meta-analysis, facilitating the extraction of multiple effect sizes from the same study cohort. This unique methodological approach allows us to examine impact of sarcopenia on all to this point researched outcome factors in HNC patients, thereby advancing our understanding of its predictive and prognostic significance [11].

This understanding of the impact of sarcopenia is crucial as it presents several potential benefits, particularly in enabling personalized patient care. To this end, multiple randomized controlled trials that modify treatment for sarcopenic patients are ongoing, including CISLOW and PECTORALIS [12, 13]. Additionally, sarcopenia can be regarded as a disease in its own right, warranting treatment. Randomized controlled trials have demonstrated significantly improved outcomes in patients who undergo prehabilitation prior to cancer surgery [14, 15]. Understanding the effects of sarcopenia and its effects on multiple patient outcomes is a critical first step.

## Methods

The systematic literature review and meta-analysis was reported according to the Preferred Reporting Items for Systematic Reviews and Meta-Analysis (PRISMA) guidelines with the checklist available in Supplementary Data S1 [16].

### Search strategy

A search strategy was developed in collaboration with a clinical librarian to search PubMed, Embase and Cochrane and was performed in November 2021, with a supplementary search performed in June 2023. The search string included studies in patients with HNC where SMM was measured using CT and/or MRI imaging. As we aimed to include all available outcome data, no specific outcome was defined in the search strategy. The complete search strategy is available in Supplementary Data S2.

### Study selection

Study selection was conducted by four authors (HvH, MvB, AS and JS). The initial screening was based on the title and abstract of each article, followed by a full-text screening. After the removal of duplicates all articles were screened by two authors independently. Any conflicts in the study selection process were resolved through consensus discussion. Studies reporting SMM measurements taken on CT or MRI and used as a predictive or prognostic factor in HNC patients were included. When multiple articles described the same population or had significant overlap the article using the larger sample size was used and the smaller one excluded. Conference abstracts and review articles were excluded. Only studies that stratified patients in sarcopenic vs. non-sarcopenic groups could be included (I.e. studies that used SMM or SMI as a continuous variable were excluded). Studies were eligible for inclusion if they included a time-to-event analysis (i.e. survival analysis, including reporting of a HR) or a comparison of sarcopenic and non-sarcopenic patients (i.e. ORs or 2 × 2 tables) or when they provided enough information where such values could be independently calculated.

### Critical appraisal

Risk of bias of included studies was assessed using the Quality In Prognosis Studies (QUIPS) tool [17]. Articles were scored across six domains: study participation, study attrition, prognostic factor and outcome measurement, study confounding and statistical analysis and reporting. Studies were scored on three to five subdomains resulting in a “low”, “moderate” or “high” risk assessment. Each of the six domains within the Quality In Prognosis Studies (QUIPS) tool was evaluated for risk of bias, categorized as low (0), moderate (1), high (2), or unknown (NR). Points were allocated based on the fulfillment of two to four criteria per domain. Articles were generally classified as low risk of bias if they enrolled a sufficient number of patients and provided adequate baseline characteristics encompassing various factors such as sex, age, tumor localization, and stage, as well as treatment details. Additionally, studies were required to report the imaging modality used and the chosen level of measurement. Studies disclosing loss to follow-up were deemed to have the lowest risk of bias in the study attrition domain. All articles were screened by two authors independently. Disagreements in domain scoring were resolved through consensus discussions, with input from a third author if necessary. Results were displayed in a “traffic light” plot generated using *robvis*, a web-based application [18].

### Data synthesis and analysis

One author (HvH) extracted the data from the included articles. Extracted data includes: First author’s name, year of publication, continent and country of population studied, sample size, age, sex, BMI, tumor staging, number of sarcopenic patients, level of measurement, imaging modality used, the origin of the cut-off value that was used in the study, whether the cut-off was sex-specific, treatment modality, outcome type and the associated Odds Ratio (OR) for risk-of-event outcomes or Hazard Ratio (HR) for time-to-event outcomes and the variance. When an OR was not provided in a study but other data such as a 2 × 2 table was available, the OR and associated variance was calculated.

### Multilevel approach

A majority of the included studies reported on multiple outcome measures. Each individual outcome measure was extracted separately. This violates the assumption of individual effect sizes in a meta-analysis. To overcome this, and at the same time provide an overall view of the effect of sarcopenia on HNC patients, a multilevel approach was used. Multilevel approaches can solve this problem, so that multiple effect sizes within a study may be included, while controlling for dependency [19, 20]. A three-level approach was used. Level 1 represents the sampling variance for each effect size. Level 2 represents the variance within studies and level 3 represents the variance between studies. A random effects model was applied using R.

## Results

### Literature search results and study characteristics

**Fig. 1:**
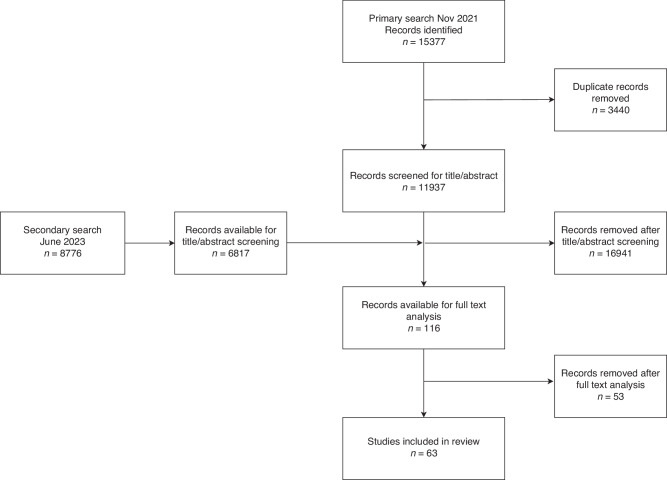
Flowchart of search strategy. This flow diagram illustrates the systematic review search process, detailing the number of records identified, screened, included, and excluded.

Literature was searched until June 2023 and 63 articles were included for analysis. Figure 1 shows a flowchart of the search results. Table 1 shows an overview of the analyzed studies.

**Table 1:** Overview of studies.

Author, year, country	Age, ± SD or Median [IQR] (range)	Total patients	Male (%)	Imaging modality used and level of measurement	Study design	Cut-off value	Origin of cut-off value	Sarcopenia prevalence (%)	Outcomes
Achim, 2017 [21], USA	66.0 ± 9.0	70	88.0	CT, L3	RCS	<52.4 cm^2^/m^2^ M <38.5 cm^2^/m^2^ F	Literature	77.14	Complications
Ahern, 2023 [22], Australia	61.2 ± 9.7	101	91.0	CT, L3	OBS	<41 F/<43 M, <53 cm^2^ obese patients	Literature	66.3	OS
Alwani, 2020 [23], USA	59.4 ± 13	168	73.2	CT, L3	RCS	<32.0 cm^2^/m^2^ F, <41.6 cm^2^/m^2^ M	Literature	27.3	Complications
Ansari, 2020 [24], Netherlands	62.4 ± 10.2	78	69.2	CT, C3	RCS	<43.2 cm^2^/m^2^ LSMI	Literature	61.5	OS, DFS
Ansari, 2022 [25], Netherlands	66.7 ± 12.5	53	61.9	CT, C3	RCS	<43.2 cm^2^/m^2^ LSMI	Literature	79.2	Complications
Arribas, 2021 [26], Spain	57.7 ± 9.62	61	85.2	CT, L3	RCS	<52.4 cm^2^/m^2^ M < 38.5 cm^2^/m^2^ F	Literature	48.2	OS, PFS
Bardoscia, 2022 [27], Italy	64.5, (56.3–72.35)	225	75.6	CT,L3	RCS	<41 cm^2^/m^2^ F, <43 cm^2^/m^2^ M or <53 cm^2^/m^2^ obese patients	Literature	23.3	OS, DSS, PFS
Becker, 2023 [28], Switzerland	NR	97	80.4	CT, C3	RCS	<43.2 cm^2^/m^2^ LSMI	Literature	43.3	CDLT, complications
Bentahila, 2022 [29], France	60.4 ± 9.4	212	83.0	CT, L3	RCS	SMI < 43.3 cm^2^/m^2^ M < 33.09 cm^2^ /m^2^ F	Literature	25.5	OS, DFS
Bonavolanta, 2023 [30], Italy	73 ± NR	426	56.0	CT, C3	RCS	<43 M cm^2^ < 41 cm^2^ M obese, <41 cm^2^ F	Literature	58.7	OS
Bril, 2019 [31], Netherlands	64.7 ± 9.1	235	82.1	CT/MR C3	RCS	<43.2 cm^2^/m^2^	Statistics	46.4	OS, Complications
Bril, 2022 [32], Netherlands	59.9 ± 6.7	153	73.2	CT, C3	RCS	<13.1 cm^2 ^M, <10.7 cm^2 ^F	Statistics	54.9	OS
Casayas, 2022, [33] Spain	65.7 ± 10.3	86	100	CT, C3	RCS	≤35.5 cm2	Statistics	46.5	Complications
Chang, 2021 [34], Taiwan	NR	125	90.4	CT C3	RCS	<20.71 cm^2^/m^2^	Statistics	36.9	OS, DFS
Chargi, 2019 [35], Netherlands	81.5 ± 6.5	85	44.7	CT C3	RCS	<43.2 cm^2^/m^2^ LSMI	Literature	48.2	OS
Chargi, 2020 [36], Netherlands	NR	216	66.2	CT/MR C3	RCS	OS < 43.0 cm^2^/m^2^, DFS < 43.2 cm2/m2	Literature	64.8	OS, DFS
Chargi, 2022 [37], Netherlands	57.7 ± 8.4	343	68.5	CT/MR C3	RCS	<43.2 cm^2^/m^2^	Literature	58.0	CDLT
Choi, 2020 [38], Korea	58.5 ± 12.8	79	82.3	CT C1 TH1	RCS	<605.77 cm^3 ^M, <445.42 cm^3 ^F	Statistics	13.9	OS, RFS
Erul, 2023 [39], Turkey	NR	123	89.4	CT C3	RCS	<52.4 cm^2^/m^2^ M, <38.5 cm^2^/m^2^ F	Literature	57.0	OS, DFS
Findlay, 2020 [40], Australia	61.0 ± 11.6	79	82.3	CT L3	RCS	<41 cm^2^/m^2^ F, <43 cm^2^/m^2^ M or <53 cm^2^/m^2^ obese patients	Literature,	53.1	OS
Findlay, 2021 [41], Australia	60.0 ± 13.0	277	78.0	CT L3	RCS	<41 cm^2^/m^2^, <43 cm^2^/m^2^ M or <53 cm^2^/m^2^ obese	Literature	52.3	OS
Galli, 2022 [42], Italy	65.9 ± 12.2	65	81.0	CT/MR C3	RCS	<11.25 cm^2^/m^2^ or <34.91 cm	Statistics	40.0 47.6	Complications
Ganju, 2019 [43], USA	60 (19–88)	246	87.4	CT C3	RCS	<43 cm^2^/m^2^ <53 cm^2^/m^2^ M, <41 cm^2^/m^2^	Literature	41.9	OS, PFS
Grossberg, 2016 [44], USA	57.7 ± 9.4	190	84.2	CT L3	RCS	<52.4 cm2 M, <38.5 cm^2 ^F,	Literature	35.3	OS, LRC
Haehl, Germany, 2022 [45]	NR	280	NR	CT C3	RCS	45.5 cm^2^/m^2^ M, <34.3 cm^2^/m^2^ F	Statistics	55.3	OS
He, 2020 [46], China	NR	1767	78.2	CT L3	RCS	<52 cm^2^ /m^2^ M <38 cm^2^ /m^2^ F. BMI ≥ 30 kg/<54 cm^2^/m^2^ for men and <47 cm^2^/m^2^ for women.	Literature	38.7	OS
Hua, 2020, China	45 (18–84)	862	74.2	CT C3	RCS	<18.82 cm^2^/m^2^	Statistics	19.7	DMFS, Complications, OS
Hua, 2021 [47], China	45 (18–84)	806	74.7	CT C3	RCS	<22.0 M cm^2^/m^2^ <18.6 F cm^2^/m^2^	Literature	24.4	OS
Huang, 2019 [48], China	46 NR	394	75.6	CT L3	RCS	<40.8 cm^2^/m^2^ M <34.9 cm^2^/m^2^ F	Literature	33.0	OS
Huang, 2021, [49] China	45.7 ± 10.4	82	67.2	CT L3	PROS	<40.8 cm^2^/m^2^ M <34.9 cm^2^/m^2^ F	Literature	45.0	CDLT
Huang, 2022 [50], China	54.2 ± 11.0	592	87.5	CT C3	RCS	<46.7 cm^2^/m^2^ M <30.3 cm^2^/m^2^ F	Literature	53.2	Complications
Huiskamp, 2020 [51], Netherlands	62.2 ± 7.2	91	64.7	CT/MR C3	RCS	<45.2 cm^2^	Literature	74.7	CDTL, DFS, OS
Jin, 2022 [52], USA	59	52	85	CT C3	RCS	<9.3 mm^2^/m^2^	Statistics	58.8	PFS, OS
Jones, 2021 [53],USA	60.4 ± 13.7	239	68.2	CT L3	RCS	<41.6 M cm^2^/m^2^ <32.0 F cm^2^/m^2^	Literature	25.9	Intraoperative Blood transfusions
Jung, 2019 [54], Korea	64 [56–73]	305	87.2	CT C3/L3	PROS	<174.5 cm^2 ^M <56.3 cm^2 ^F	Statistics	50.0	OS
Jung, 2019 [55], Korea	64 [56–73]	258	86.4	CT L3	PROS	<52.4 cm^2^/m^2^ M <38.5 cm^2^/m^2^ F	Literature	6.6	DFS, OS
Jung, 2021 [56], Korea	71.9 ± 5.1	190	82.1	CT L3	PROS	<52.4 cm^2^/m^2^ M <38.5 cm^2^/m^2^ F	Literature	33.7	Complications, OS, Hospital readmissions
Karsten, [57] Netherlands, 2019	59 ± 7	128	70.0	CT C3	RCS	<12.7 cm^2^/m^2^	Statistics	57.0	Feeding tube dependency
Lee, 2021 [58], Taiwan	51 [45–58]	174	91.4	CT C3	RCS	<52.4 cm^2^/m^2^ M <35.2 cm^2^/m^2^ F	Statistics	32.8	OS, DFS, Complications
Lee, 2021 [59], Korea	68.8 ± 2.8	106	79.2	CT and MR Sylvian fissure	RCS	<6.24 mm	Statistics	50	PFS
Lee, 2021 [60], Taiwan	51 [45–58]	155	92.3	CT C3	RCS	<57.4 cm^2^/m^2^ M <48.0 cm^2^/m^2^ F	Statistics	25	OS, RFS
Lin, Taiwan, 2019	55.0 ± 12.0	276	92.4	CT C3	RCS	<47.5 cm^2^/m^2^	NR	NR	DSS, OS
Makiguchi, 2019 [62], Japan	60.3 ± 11.2	120	67.2	CT L3	RCS	<36.02 cm^2^/m^2^ M <31.76 cm^2^/m^2^ F	Statistics	25.4	Complications
Mascarella, 2022 [63], USA	61.8 ± 14.1	127	67.0	CT C3	PROS	<1100 mm/m^2^ M <880 mm/m^2^ F	Statistics	NR	Hospital stay, OS, Complications
Mascarella, 2022 [64], USA	62.4 ± 14	127	67.7	CT C3	PROS	NR	NR	30.7	CDLT
McGoldrick, 2022 [65], UK	74, (65–92)	111	69.0	CT C2	RCS	NR	Statistics	33	OS
Morse, 2022 [66], USA	NR	272	81.9	CT C3	RCS	NR	Statistics	50	CDLT, Complications
Nagpal, 2021 [67] India	60.4 NR	306	88.0	CT C3	RCS	<32 cm^2^/m^2^	Statistics	50	Disease control
Nishikawa, 2018 [68], Japan	66 (28–89)	85	78	CT L3	RCS	<46.7 cm^2^/m^2^ M, <30.3 cm^2^/m^2^ F	Statistics	46	OS
Pai, 2018 [69], Taiwan	51.4 ± 11.3	881	82.7	CT T2	RCS	<51.7 cm^2^/m^2^ M, <34.3 cm^2^/m^2^ F	Statistics	50	DMFS, LRG, OS
Rijn-Dekker, 2020 [70], Netherlands	NR	744	74.2	CT C3	RCS	<42.4 cm^2^/m^2^ M, <30.6 cm^2^/m^2^ F	Statistics	25	DFS, OS
Shodo, 2021 [71], Japan	64.2 ± 8.3	41	100	CT L3	RCS	<39.7 cm^2^/m^2^	Statistics	26.8	CDLT
Stone, 2019 [72], USA	61.1 ± 11	260	74.2	CT L3	RCS	<52.4 cm^2^/m^2^ M, 38.5 cm^2^/m^2^ F	Literature	55.4	DFS, OS
Takenaka, 2022 [73], Japan	65 (23–80)	114	74.6	CT L3	RCS	<39.84 cm^2^/m^2^ M, 35.92 cm^2^/m^2^ F	Statistics	NR	OS, PFS
Tamaki, 2019 [74], USA	NR	113	69.9	CT L3	RCS	<43 cm2/m2 M, <41 cm^2^/m^2^ F	Literature	28.3	DFS, OS
Thureau, 2021 [75], France	61.1 ± 9.0	243	77.0	CT L3	PROS	< 52.4 cm^2^/m^2^ M, <38.5 cm^2^/m^2^ F	Literature	36.7	DFS, OS
van Heusden, 2022 [73], Netherlands	61.8 [56.0–67.4]	99	72.7	CT C2, L3, C3	RCS	C2: NR L3: < 43.2 cm^2^/m^2^	C2: Statistics L3: Literature	C2: 25 L3: NR	OS
Wendrich, 2017 [77], Netherlands	54.5 ± 9.4	112	64.3	CT C3	RCS	<43.2 cm^2^/m^2^	Statistics	54.5	CDLT
Willemsen, 2023 [78], Netherlands	63.2 ± 8.0	98	85.0	CT L3	RCS	<43 cm^2^/m2 M, <41 cm^2^/m^2^ F	Literature	53	OS, PFS
Xing, 2021 [79], Netherlands	46.5 (23–70)	96	75.0	CT L3	RCS	<52.7 cm^2^/m^2^	Statistics	NR	Complications
Yamahara, 2021 [80], Japan	72 (41–92)	164	87.2	CT C3	RCS	<43.2 cm^2^/m^2^	Literature	52.4	DFS, OS, locoregional control
Yoshimura, 2020 [81], Japan	NR	103	59.2	CT L3 psoas	RCS	<6.05 cm^2^/m^2^ M, <5.097 cm^2^/m^2^ F	Statistics	28.1	DSS
Yunaiyama, 2022 [82], Japan	NR	101	85	CT hyoid level	RCS	<16.88 cm^2^/m^2^	Statistics	55.4	OS

### Quality assessment

**Fig. 2:**
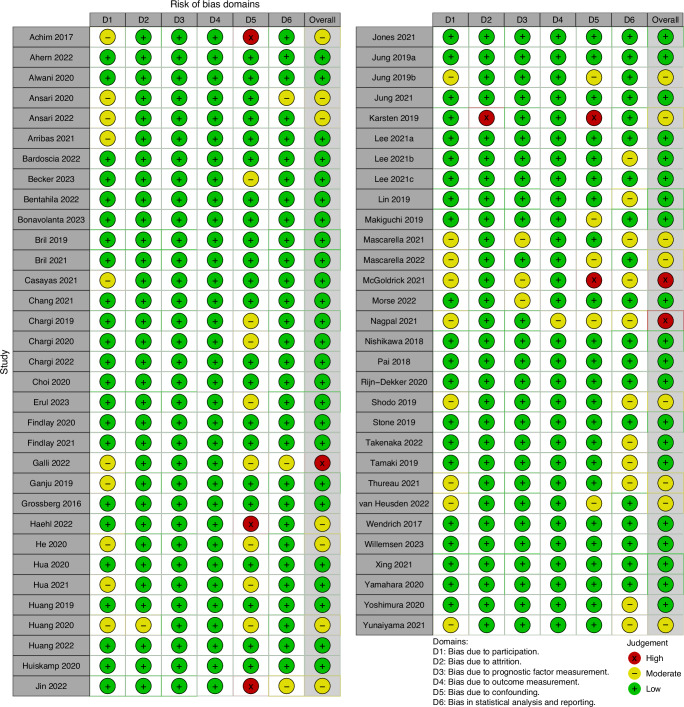
Stoplight table of QUIPS. D1: bias due to participation, D2: bias due to attrition, D3: bias due to prognostic factor measurement, D4: bias due to outcome measurement, D5: bias due to confounding, D6: bias due in statistical analysis and reporting. Green denotes low risk of bias, yellow moderate risk of bias and red high risk of bias.

Figure 2 shows a traffic light table of all analyzed studies. Fourteen (22.2%) articles were deemed as moderate risk of bias, with three (4.8%) articles being designated as incurring a high risk of bias. Studies scored a moderate risk of bias on the first domain due to a low number of participants or due to missing information describing the study cohort. Studies generally accounted for confounding by including covariates that are known to be associated with the studies outcomes.

### Patient population

A total of 173 effect sizes from 63 studies were extracted [21–82]. The following outcome measures were encountered: overall survival (OS), disease free survival (DFS), progression free survival (PFS), disease specific survival (DSS), locoregional control (LRC), distant metastasis free survival (DMFS), recurrence free survival (RFS), surgical-treatment-related complications, non-surgical treatment-related complications, chemotherapy dose-limiting toxicity (CDLT), feeding tube dependency, hospital readmission and intraoperative blood transfusions.

As described above risk-of-event outcomes and time-to-event data were analyzed separately. We included 70 HRs from 39 studies and 103 ORs from 34 studies. In total, these studies provide data from 14,804 HNC patients with sample sizes ranging from 41 to 1767 patients. Studies were performed in Asia (*n* = 25), Europe (*n* = 25), North America (*n* = 10) and Australia (*n* = 3). No studies from South-America or Africa were retrieved. Seven (11.1%) of these studies were prospective while the rest were retrospective in design. The patients had a mean age of 61.3 years (*sd* = 6.9) and a mean BMI of 24.4 kg/m² (*sd* = 1.50). Most studies included patients with advanced stage HNC: the mean percentage of patients with stage III/IV disease was 78.6% (*sd* = 21.0%). Studies include only patients treated with chemoradiotherapy (*n* = 13), surgery only (*n* = 5), or surgery with adjuvant radiotherapy (*n* = 4). There were 39 studies that included all their patients in a single cohort regardless of the therapy. Two studies described patients treated with immunotherapy with or without other treatment. A variety of cut-off values were employed across the studies. Twenty-eight (46.6%) studies determined the cut-off value within their cohort, while the remaining 35 utilized literature-sourced values, with a LSMI of <43.2 cm²/m² being the most commonly cited. For studies using lumbar measurements, almost all quantified all skeletal muscles at L3, with the exception of one study that focused solely on the psoas muscle area. In studies measuring SMM at C3, most adopted the method pioneered by Swartz et al., which involves measuring the paravertebral and sternocleidomastoid (SCM) [83]. If one SCM is affected by prior surgery or disease, the unaffected SCM area is doubled. Two studies using the C3 landmark excluded the SCM area, and one study quantified cervical muscle volume rather than area. Additionally, two studies included measurements at the second cervical vertebra (C2), focusing on the masticatory muscles. One study assessed muscle area at the hyoid level, quantifying the infrahyoid skeletal muscle area while excluding the pharyngeal constrictor and trapezius muscles.

Sarcopenia was determined on CT imaging in 55 studies. In the remaining eight studies CT and MRI were both used, depending on which scan was present. None of the included studies utilized MRI exclusively to evaluate skeletal muscle mass. The level of measurement was cervical in 36 studies, lumbar in 24 studies, thoracic in one study, cervical or lumbar (whichever was present) in one study and cranial in one study. Twenty-two studies used a transformation formula to estimate the SMM on L3 using a cervical scan as described in Swartz et al. [83]. On average 44.4% of patients was sarcopenic *(sd* = 15.6).

### Risk of event (OR) analysis

#### Full model

A total of *k* = 103 effect sizes were included in this analysis. There was significant heterogeneity between all effect sizes (*Q* (102) = 419.418, *p* < 0.001). The total variance was 0.305, of which 0.041 was sampling error variance and 0.264 could not be attributed to sampling error. The distribution of the variance was 13.5% at level one (population variance), 32.2% at level two (within-study variance) and 54.4% at level three (between-study variance). The Akaike’s Information Criterion (AIC, a measure of the model fit, with lower values indicating better fit) of the three-level model was 231.8. Constraining the variance at level two to 0 increased the AIC to 241.4 (*p* < 0.001). Constraining the variance at level three to 0 significantly increased the AIC to 241.4 (*p* < 0.001). These findings confirm that a three level model is the optimal model for this dataset.

#### Analysis per predictive and prognostic outcome

Please note that the described outcomes are *log OR*, where values above 0 imply worse outcomes for sarcopenic patients and negative values 0 imply better outcomes for sarcopenic patients. The estimated log OR of the whole dataset was 0.644 (95% CI = 0.505–0.783, *p* < 0.001), suggesting that patients with sarcopenia have a higher risk of worse outcomes (Table 2). There were sixteen effect sizes on chemotherapy dose limiting toxicity (CDLT), showing that sarcopenia is a risk factor for dose limiting toxicity (log OR 0.760, 95% CI = 0.277–1.243, *p* < 0.001). There were 69 effect sizes on complications, showing that sarcopenia is a risk factor for complications (log OR = 0.669, 95% CI = 0.441–0.897, *p* < 0.001). There were 8 effect sizes for disease control, showing that sarcopenia was a risk factor for worse disease control (log OR = 0.638, 95% CI = 0.161–1.114, *p* = 0.02). There were two effect sizes for hospitalization readmission. There was no significant effect of sarcopenia on hospitalization outcomes, with a log OR of 0.683 (95% CI = −3.051–4.417, *p* = 0.26). There were seven effect sizes for survival outcomes. All seven studies described overall survival as a 1-, 2- or 5-year surviving fraction and were therefor included in this risk-of-event analysis. There was a significant effect of sarcopenia on survival, with a log OR of 0.808 (95% CI = 0.509–1.107, *p* < 0.001).

**Table 2:** Effect sizes of sarcopenic vs. non-sarcopenic patients per outcome.

Analysis	Log OR	95% CI	SE	t (df)
Overall	0.644	0.505–0.784	0.070	9.185 (102)
CDLT	0.760	0.277–1.243	0.227	3.353 (15)
Complications	0.634	0.413–0.854	0.110	5.740 (68)
Disease control	0.547	0.117–0.977	0.182	3.005 (7)
Hospitalization	0.683	−3.051–4.417	0.294	2.325 (1)
Survival	0.808	0.509–1.107	0.122	6.613 (6)

*CDLT* Chemotherapy dose limiting toxicity.

#### Moderator analysis

Several variables were investigated as a moderator, including the imaging modality. The only modalities that had been performed in the studies were CT imaging and CT or MR whichever was available. This variable was not a significant moderator (Test of Moderators F (1,101) = 0.108, *p* = 0.743), suggesting that the effect of sarcopenia on outcome is not influenced by the type of imaging that was used. The level of measurement (I.e. cervical or lumbar measurements) was not a significant moderator (F (2,100) = 0.627, *p* = 0.536). The use of a prediction rule to estimate the skeletal muscle index (SMI) at the L3 level, versus using another level was also not a significant moderator (F (1,101) = 2.938, *p* = 0.090). The type of therapy that patients received was not a significant moderator (F (3,99) = 0.457, *p* = 0.713). The following variables were no significant moderators as well: continent of study (F (3,99) = 0.953, *p* = 0.418), BMI (F (1,52) = 0.088, *p* = 0.768), age (F (1,72) = 0.874, *p* = 0.353), gender (F (1,101) = 0.961, *p* = 0.329) and stage (F (1,95) = 0.095, *p* = 0.758).

### Time-to-event (HR) analysis

#### Full model

A total of *k* = 70 effect sizes were included in these analyses. There was significant heterogeneity between effect sizes (Q (69) = 612.164, *p* < 0.001*)*. The total variance at level two (within study variance) was 0.267 and the variance at level three (between study variance) was 0.019. The I² at level 1 was 8.42%, the variance at level 2 was 6.09% and the variance at level 3 was 85.48%. The AIC of the three-level model was 102.95. Constraining the variance at level three to zero significantly deteriorated the model fit (AIC = 126.6, *p*-value for change <0.001). Constraining the variance at level two to zero deteriorated the model fit (increased the AIC) to 103.7, but this was not a significant increase (*p* = 0.10). In this analysis, the three-level model does not offer a statistically significant improvement of the model fit. However, it is unwanted in meta-analysis to treat related outcomes (i.e. OS or DFS outcomes obtained from the same patient sample) as independent. Therefore, the three-level structure was retained.

The pooled log HR for the effect of sarcopenia on time-to-event outcomes was 0.606 (95% CI = 0.422–0.791, *p* < 0.001), suggesting that patients with sarcopenia had reduced time-to-event outcome. The only available time-to-event outcome categories were disease control (locoregional control, local disease control) and survival outcomes (overall survival, progression free survival, regression free survival, disease free survival, distant metastasis free survival). There were 27 effect sizes on disease control outcomes. Sarcopenic patients were at significantly higher risk of worse disease control with a log HR of 0.544 (95% CI 0.296–0.792, *p* < 0.001). There were 43 effect sizes on survival, with a log HR of 0.674 (95% CI 0.482–0.866, *p* < 0.001).

#### Moderator analysis

Moderator analysis showed that imaging modality (CT or CT/MRI) were not a significant moderator (F (1,8) = 0.3612, *p* = 0.062), nor was the use of a prediction rule (F (1,67) = 0.493, *p* = 0.485). Interestingly, the level of measurement was a significant moderator (F (2,67) = 4.369, *p* = 0.016). This was mostly influenced by the single study that used cranial level imaging to determine sarcopenia [59]. There was no significant difference between cervical or lumbar levels of measurement (*p* = 0.289). Continent of study was not a significant moderator (F (3,66) = 2.123, *p* = 0.106), nor was BMI (F (1,26) = 0.061, *p* = 0.807), age (F (1,34) = 0.642, *p* = 0.429), sex (F (1,66) = 1.750, *p* = 0.190), stage (F (1,46) = 0.323, *p* = 0.323) or treatment (F (4,65) = 0.912, *p* = 0.462).

### Funnel plot

**Fig. 3:**
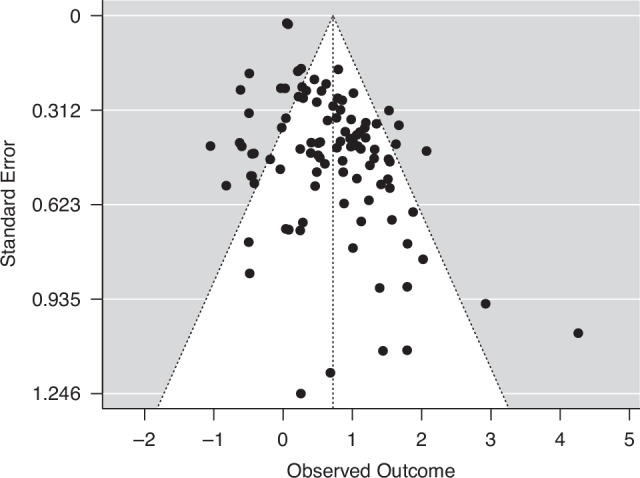
Funnel plot for publication bias in articles reporting OR in head and neck cancer patients.

**Fig. 4:**
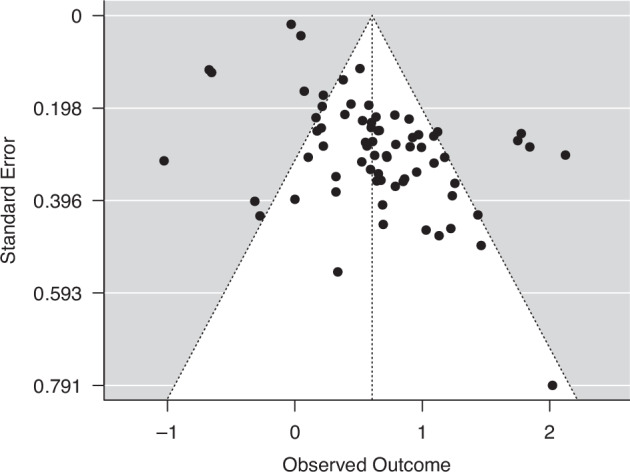
Funnel plot for publication bias in articles reporting HR in head and neck cancer patients.

To investigate publication bias, funnel plots were established (Figs. 3 and 4). These plots suggested that no relevant publication bias was present.

## Discussion

Our systematic review and meta-analysis describes data from 14,804 patients across 63 studies and shows a significant association between sarcopenia and several adverse clinical outcomes in patients with HNC. Specifically, sarcopenia was correlated with diminished survival rates and reduced disease control. Moreover, there were higher rates of complications and CDLT. It should be noted that the included studies exhibited considerable variability in disease stage, cancer site, and treatment modalities. Moreover, there was notable heterogeneity in the definition of sarcopenia across studies, including variations in measurement level, cut-off thresholds, and imaging modalities utilized. Regardless, our multilevel analysis demonstrated that sarcopenia is a consistently significant predictive and prognostic factor across all patient groups. This study highlights the potential role of sarcopenia in pre-treatment risk stratification for HNC patients, irrespective of treatment type, patient-specific factors, or tumor characteristics.

Sarcopenia is defined as the lack of SMM and function and was previously thought to be a condition exclusively related to aging [84]. However, sarcopenia can develop in cancer patients as the result of chronic systemic inflammatory processes as a reaction to the tumor [7]. Skeletal muscle tissues act as an endocrine organ secreting specific cytokines, referred to as myokines [85]. These myokines impact tissue repair and immune regulation and surveillance. A lack of SMM results in fewer myokines and therefore decreased immune function and tissue repair [85]. In HNC-patients, tumors arise in the upper air- and foodway. This may result in dysphagia or odynophagia, causing malnutrition and a catabolic state compounding the tumor biochemical related systemic negative effects [86–88]. These adverse effects are not confined to late disease stages, as research indicates that a significant proportion of patients present with some degree of malnutrition [89]. Within the realm of HNC, the prevalence of sarcopenia is notably elevated, with our analysis showing a sarcopenia prevalence of 44.4% among included patients, surpassing rates observed in other cancer types and corroborating earlier findings [90]. Based on its definition sarcopenia is diagnosed by measuring SMM and its function [7]. Muscle function, which can be determined by e.g. hand grip strength or gait speed, was rarely measured in the included studies, probably as it cannot be determined retrospectively. There is significant heterogeneity in the literature regarding the usage of terms and “sarcopenia” and “low muscle mass”, and these terms have been used interchangeably although this is technically incorrect. Nevertheless, low SMM by itself has been shown to be a significant predictor in earlier research and most clinical research prioritizes the amount of SMM [91, 92]. There is no current gold standard for the analysis of body composition although the reference standard for determining the amount of SMM is dual-energy x-ray absorptiometry, also referred to as DEXA, which is seldom available in a routine clinical setting [93]. Alternatives have been proposed in the form of MRI, CT, body impedance analysis and ultrasound. SMM measured on a cross-sectional image at the level of the third lumbar vertebra was first shown to correlate closely with whole-body muscle mass and has been used as a clinical standard since [9]. Additionally, research has identified surrogate measurement methods at cervical and thoracic levels, facilitating broader applicability in muscle mass assessment [83, 94]. Decreased SMM measured on cross-sectional imaging is categorized as radiologically defined sarcopenia, serving as a distinct classification from sarcopenia. However, due to ease of access most clinical research has focused on radiologically defined sarcopenia even to such a degree that the terms are used interchangeably. There are variations in regard to every step in the methodology of measuring SMM. For instance, there are several different software applications that allow for detailed muscle quantification. Previous research by van Vugt et al. showed that were was excellent inter- and intra-observer agreement in the measurement of SMM at an abdominal level using four common software applications [95]. With regards to chosen modality, research has shown that there is excellent agreement between muscle measurements taken on CT and MRI which is corroborated by our findings [96, 97]. Our analysis shows that CT emerged as the most commonly used modality for analyzing SMM, with the vast majority of studies analyzing CT imaging. This preference is likely driven by the widespread availability of CT imaging, enabling its straightforward integration into research protocols. Additionally, the delineation of muscle areas based on Hounsfield Unit pixel values allows for convenient and accurate measurements. Regarding the level of measurement, C3 was the most frequently used, closely followed by L3, with generally consistent measurement methods applied at each respective level. One study used a different method for lumbar measurements, and five studies employed alternative methods for cervical measurements. Measurements taken at different levels were not significant moderators of effect sizes. There is notable heterogeneity in the cut-off values used across studies, with nearly half (46.6%) employing a cut-off value established within their specific cohort. While studies using literature-based cut-off values also exhibited some variation, it was considerably less pronounced. Additionally, 54.0% of the included studies applied sex-specific cut-off values. The optimal method for defining sarcopenia in HNC remains unclear, as universal, ethnicity-specific, sex-specific, and BMI-dependent criteria have all demonstrated clinically significant results [77, 98, 99]. Without access to the raw data from each individual study, it is not possible to draw definitive conclusions about the impact of the chosen cut-off values. Additional differences between studies included whether SMM was used directly, adjusted for body height, or converted to muscle mass at L3, which is currently the gold clinical standard [91]. Nevertheless, our modification analysis revealed no statistically significant changes in the results, reinforcing the robust prognostic significance of sarcopenia. These differences do highlight the lack of consensus in regards to the methods for diagnosing sarcopenia in HNC patients. Considering sarcopenia is used as a determinant in randomized controlled trials in HNC patients, it is vital to the reproducibility of these trials and other research that a “ gold standard” method is reached and applied [12, 13]. Furthermore, all included studies rely on human-driven methods to quantify SMM, although recent research has begun exploring the use of machine learning models. [100, 101]. These artificial intelligence-driven approaches eliminate the need for time-consuming measurements. Although such methods greatly facilitate further research, a standardized model has yet to be established. Consensus in regards to method and cut-off value could greatly enhance the applicability of these AI-driven methods, and prevent redundant research where different AI-models utilize different methods and hamper comparability.

Our study boasts several strengths stemming from our unique methodological approach. Previous systematic reviews have explored various outcomes related to sarcopenia in HNC patients such as OS, DFS, DSS, and PFS [102–109]. These previous meta-analyses are relatively limited because they focus on specific patient groups and treatment combinations, limiting their generalizability. In contrast, our study provides a comprehensive global overview by incorporating a broader range of data and analyzing effect sizes that were previously unreported. A standard meta-analysis averages reported effect sizes from included studies under the assumption of independence, which is often not the case. Dependency can arise internally or externally within a single study. External factors may include studies conducted by the same research groups or in similar geographical locations. Internally, dependency can manifest when certain study participants are predisposed to particular outcomes, potentially leading to associations with other closely related outcome factors. For instance, a patient with a short OS may also exhibit a shortened DFS and increased risk of complications. In such cases, using both OS and DFS outcomes for a single patient in a meta-analysis effectively duplicates the data. Moreover, studies reporting multiple effect sizes can disproportionately influence the meta-analysis. Traditionally, dependency issues are addressed by either ignoring them, extracting only one effect size per study or study sample, or utilizing statistical methods to model the dependence. Our approach tackles dependency concerns by employing the three-level or multilevel method. This approach allows us to correct for the heterogeneities and dependencies present in the gathered data, thereby offering a comprehensive analysis.

We acknowledge several limitations inherent to our study. Firstly, the predominance of retrospective cohort studies (88.9%) included in our analysis introduces potential biases inherent to this study design. The exclusion of certain studies and effect sizes due to inadequate reporting of outcomes, such as odds ratios, hazard ratios, or surrogate measures, coupled with the selective reporting of statistically significant values or singular p-values in some articles, marginally reduced our statistical power. Finally, a small subset of three studies included for analysis exhibited a high risk of bias.

## Conclusion

The results of this study show that sarcopenia as is robust biomarker for various predictive and prognostic outcomes in HNC patients. This relation was found regardless of factors such as age, BMI, tumor size or treatment. Our findings underscore the importance of integrating sarcopenia assessment into the development of personalized treatment strategies for all patients with head and neck cancer. Furthermore, our research provides a comprehensive overview of all the methods used of diagnosing sarcopenia in head and neck cancer. This will assist future research in determining the optimal method going forward.

## Supplementary information

PRISMA

search strategy

## References

[1] Gormley M, Creaney G, Schache A, Ingarfield K, Conway DI. Reviewing the epidemiology of head and neck cancer: definitions, trends and risk factors. Br Dent J. 2022;233:780–6. 10.1038/s41415-022-5166-x. 36369568 10.1038/s41415-022-5166-x PMC9652141

[2] Chow LQM. Head and neck cancer. N Engl J Med. 2020;382:60–72. 10.1056/nejmra1715715. 31893516 10.1056/NEJMra1715715

[3] Alshadwi A, Nadershah M, Carlson ER, Young LS, Burke PA, Daley BJ. Nutritional considerations for head and neck cancer patients: a review of the literature. J Oral Maxillofac Surg. 2013;71:1853–60. 10.1016/j.joms.2013.04.028. 23845698 10.1016/j.joms.2013.04.028

[4] Zwart AT, Van Der Hoorn A, Van Ooijen PMA, Steenbakkers RJHM, De Bock GH, Halmos GB. CT‐measured skeletal muscle mass used to assess frailty in patients with head and neck cancer. J Cachexia Sarcopenia Muscle. 2019;10:1060–9. 10.1002/jcsm.12443. 31134765 10.1002/jcsm.12443 PMC6818448

[5] Pressoir M, Desné S, Berchery D, Rossignol G, Poiree B, Meslier M, et al. Prevalence, risk factors and clinical implications of malnutrition in French Comprehensive Cancer Centres. Br J Cancer. 2010;102:966–71. 10.1038/sj.bjc.6605578. 20160725 10.1038/sj.bjc.6605578 PMC2844030

[6] Givens DJ, Karnell LH, Gupta AK, Clamon GH, Pagedar NA, Chang KE, et al. Adverse events associated with concurrent chemoradiation therapy in patients with head and neck cancer. Arch Otolaryngol Neck Surg. 2009;135:1209. 10.1001/archoto.2009.174. 10.1001/archoto.2009.174 20026818

[7] Cruz-Jentoft AJ, Bahat G, Bauer J, Boirie Y, Bruyère O, Cederholm T, et al. Sarcopenia: revised European consensus on definition and diagnosis. Age Ageing. 2019;48:16–31. 10.1093/ageing/afy169. 30312372 10.1093/ageing/afy169 PMC6322506

[8] Silva PB, Ramos GHA, Petterle RR, Borba VZC. Sarcopenia as an early complication of patients with head and neck cancer with dysphagia. Eur J Cancer Care. 2021;30:e13343. 10.1111/ecc.13343. 10.1111/ecc.13343 33043532

[9] Shen W, Punyanitya M, Wang Z, Gallagher D, St.-Onge MP, Albu J, et al. Total body skeletal muscle and adipose tissue volumes: estimation from a single abdominal cross-sectional image. J Appl Physiol. 2004;97:2333–8. 10.1152/japplphysiol.00744.2004. 15310748 10.1152/japplphysiol.00744.2004

[10] Fearon K, Strasser F, Anker SD, Bosaeus I, Bruera E, Fainsinger RL, et al. Definition and classification of cancer cachexia: an international consensus. Lancet Oncol. 2011;12:489–95. 10.1016/s1470-2045(10)70218-7. 21296615 10.1016/S1470-2045(10)70218-7

[11] Van Den Noortgate W, López-López JA, Marín-Martínez F, Sánchez-Meca J. Three-level meta-analysis of dependent effect sizes. Behav Res Methods. 2013;45:576–94. 10.3758/s13428-012-0261-6. 23055166 10.3758/s13428-012-0261-6

[12] Van Beers MA, Speksnijder CM, Van Gils CH, Frederix GWJ, Dankbaar JW, De Bree R. Prophylactic pectoralis major flap to compensate for increased risk of pharyngocutaneous fistula in laryngectomy patients with low skeletal muscle mass (PECTORALIS): study protocol for a randomized controlled trial. BMC Cancer. 2024;24:76. 10.1186/s12885-023-11773-7. 38225572 10.1186/s12885-023-11773-7 PMC10788993

[13] Schaeffers AWMA, Devriese LA, Van Gils CH, Dankbaar JW, Voortman J, De Boer JP, et al. Low dose cisplatin weekly versus high dose cisplatin every three weeks in primary chemoradiotherapy in head and neck cancer patients with low skeletal muscle mass: the CISLOW-study protocol. PLOS One. 2023;18:e0294147. 10.1371/journal.pone.0294147. 38011186 10.1371/journal.pone.0294147 PMC10681175

[14] Molenaar CJL, Minnella EM, Coca-Martinez M, Ten Cate DWG, Regis M, Awasthi R, et al. Effect of multimodal prehabilitation on reducing postoperative complications and enhancing functional capacity following colorectal cancer surgery: the PREHAB randomized clinical trial. JAMA Surg. 2023;158:572. 10.1001/jamasurg.2023.0198. 36988937 10.1001/jamasurg.2023.0198 PMC10061316

[15] Loewen I, Jeffery CC, Rieger J, Constantinescu G. Prehabilitation in head and neck cancer patients: a literature review. J Otolaryngol Head Neck Surg. 2021;50:2. 10.1186/s40463-020-00486-7. 33407922 10.1186/s40463-020-00486-7 PMC7789666

[16] Page MJ, McKenzie JE, Bossuyt PM, Boutron I, Hoffmann TC, Mulrow CD, et al. The PRISMA 2020 statement: an updated guideline for reporting systematic reviews. BMJ. 2021;n71. 10.1136/bmj.n71 10.1136/bmj.n71 PMC8005924 33782057

[17] Hayden JA, Van Der Windt DA, Cartwright JL, Côté P, Bombardier C. Assessing bias in studies of prognostic factors. Ann Intern Med. 2013;158:280. 10.7326/0003-4819-158-4-201302190-00009. 23420236 10.7326/0003-4819-158-4-201302190-00009

[18] McGuinness LA, Higgins JPT. Risk-of-bias VISualization (robvis): an R package and Shiny web app for visualizing risk-of-bias assessments. Res Synth Methods. 2021;12:55–61. 10.1002/jrsm.1411. 32336025 10.1002/jrsm.1411

[19] Geeraert L, Van Den Noortgate W, Grietens H, Onghena P. The effects of early prevention programs for families with young children at risk for physical child abuse and neglect: a meta-analysis. Child Maltreat. 2004;9:277–91. 10.1177/1077559504264265. 15245680 10.1177/1077559504264265

[20] Van Den Noortgate W, López-López JA, Marín-Martínez F, Sánchez-Meca J. Meta-analysis of multiple outcomes: a multilevel approach. Behav Res Methods. 2015;47:1274–94. 10.3758/s13428-014-0527-2. 25361866 10.3758/s13428-014-0527-2

[21] Achim V, Bash J, Mowery A, Guimaraes AR, Li R, Schindler J, et al. Prognostic indication of sarcopenia for wound complication after total laryngectomy. JAMA Otolaryngol Neck Surg. 2017;143:1159. 10.1001/jamaoto.2017.0547. 10.1001/jamaoto.2017.0547 28448668

[22] Ahern E, Brown TE, Campbell L, Hughes BGM, Banks M, Lin CY, et al. Impact of sarcopenia and myosteatosis on survival outcomes for patients with head and neck cancer undergoing curative-intent treatment. Br J Nutr. 2023;129:406–15. 10.1017/s0007114522000435. 35152926 10.1017/S0007114522000435 PMC9876810

[23] Alwani MM, Jones AJ, Novinger LJ, Pittelkow E, Bonetto A, Sim MW, et al. Impact of sarcopenia on outcomes of autologous head and neck free tissue reconstruction. J Reconstr Microsurg. 2020;36:369–78. 10.1055/s-0040-1701696. 32088918 10.1055/s-0040-1701696

[24] Ansari E, Chargi N, Van Gemert JTM, Van Es RJJ, Dieleman FJ, Rosenberg AJWP, et al. Low skeletal muscle mass is a strong predictive factor for surgical complications and a prognostic factor in oral cancer patients undergoing mandibular reconstruction with a free fibula flap. Oral Oncol. 2020;101:104530. 10.1016/j.oraloncology.2019.104530. 31881447 10.1016/j.oraloncology.2019.104530

[25] Ansari E, Chargi N, Van Es RJJ, Dieleman FJ, Van Cann EM, De Bree R. Association of preoperative low skeletal muscle mass with postoperative complications after selective neck dissection. Int J Oral Maxillofac Surg. 2022;51:1389–93. 10.1016/j.ijom.2022.02.008. 35256219 10.1016/j.ijom.2022.02.008

[26] Arribas L, Plana M, Taberna M, Sospedra M, Vilariño N, Oliva M, et al. Predictive value of skeletal muscle mass in recurrent/metastatic head and neck squamous cell carcinoma patients treated with immune checkpoint inhibitors. Front Oncol. 2021;11:699668. 10.3389/fonc.2021.699668. 34249760 10.3389/fonc.2021.699668 PMC8267860

[27] Bardoscia L, Besutti G, Pellegrini M, Pagano M, Bonelli C, Bonelli E, et al. Impact of low skeletal muscle mass and quality on clinical outcomes in patients with head and neck cancer undergoing (chemo)radiation. Front Nutr. 2022;9:994499. 10.3389/fnut.2022.994499. 36466387 10.3389/fnut.2022.994499 PMC9715267

[28] Becker JN, Hermann R, Wichmann J, Sonnhoff M, Christiansen H, Bruns F. Low skeletal muscle mass is predictive of dose-limiting toxicities in head and neck cancer patients undergoing low-dose weekly cisplatin chemoradiotherapy. PLOS One. 2023;18:e0282015. 10.1371/journal.pone.0282015. 36802403 10.1371/journal.pone.0282015 PMC9942991

[29] Bentahila R, Giraud P, Decazes P, Kreps S, Nay P, Chatain A, et al. The impact of sarcopenia on survival and treatment tolerance in patients with head and neck cancer treated with chemoradiotherapy. Cancer Med. 2023;12:4170–83. 10.1002/cam4.5278. 36263581 10.1002/cam4.5278 PMC9972161

[30] Bonavolontà P, Improta G, Dell’Aversana Orabona G, Goglia F, Abbate V, Sorrentino A, et al. Evaluation of sarcopenia and sarcopenic obesity in patients affected by oral squamous cell carcinoma: a retrospective single-center study. J Cranio Maxillofac Surg. 2023;51:7–15. 10.1016/j.jcms.2023.01.014. 10.1016/j.jcms.2023.01.014 36739189

[31] Bril SI, Pezier TF, Tijink BM, Janssen LM, Braunius WW, De Bree R. Preoperative low skeletal muscle mass as a risk factor for pharyngocutaneous fistula and decreased overall survival in patients undergoing total laryngectomy. Head Neck. 2019;41:1745–55. 10.1002/hed.25638. 30663159 10.1002/hed.25638 PMC6590286

[32] Bril SI, Al‐Mamgani A, Chargi N, Remeijer P, Devriese LA, De Boer JP, et al. The association of pretreatment low skeletal muscle mass with chemotherapy dose‐limiting toxicity in patients with head and neck cancer undergoing primary chemoradiotherapy with high‐dose cisplatin. Head Neck. 2022;44:189–200. 10.1002/hed.26919. 34713519 10.1002/hed.26919 PMC9298001

[33] Casasayas M, García-Lorenzo J, Gómez-Ansón B, Medina V, Fernández A, Quer M, et al. Low skeletal muscle mass assessed directly from the 3rd cervical vertebra can predict pharyngocutaneous fistula risk after total laryngectomy in the male population. Eur Arch Otorhinolaryngol. 2022;279:853–63. 10.1007/s00405-021-07127-3. 34665301 10.1007/s00405-021-07127-3 PMC8795024

[34] Chang S, Hsu C, Tsai Y, Chang G, Tsai M, Huang EI, et al. Prognostic value of third cervical vertebra skeletal muscle index in oral cavity cancer: a retrospective study. Laryngoscope. 2021;131:E2257–E2265. 10.1002/lary.29390. 33433021 10.1002/lary.29390

[35] Chargi N, Bril SI, Emmelot-Vonk MH, De Bree R. Sarcopenia is a prognostic factor for overall survival in elderly patients with head-and-neck cancer. Eur Arch Otorhinolaryngol. 2019;276:1475–86. 10.1007/s00405-019-05361-4. 30830300 10.1007/s00405-019-05361-4 PMC6458984

[36] Chargi N, Bril SI, Swartz JE, Wegner I, Willems SM, De Bree R. Skeletal muscle mass is an imaging biomarker for decreased survival in patients with oropharyngeal squamous cell carcinoma. Oral Oncol. 2020;101:104519. 10.1016/j.oraloncology.2019.104519. 31855705 10.1016/j.oraloncology.2019.104519

[37] Chargi N, Bashiri F, Wendrich AW, Smid EJ, De Jong PA, Huitema ADR, et al. Image-based analysis of skeletal muscle mass predicts cisplatin dose-limiting toxicity in patients with locally advanced head and neck cancer. Eur Arch Otorhinolaryngol. 2022;279:3685–94. 10.1007/s00405-021-07229-y. 35038029 10.1007/s00405-021-07229-y

[38] Choi Y, Ahn K, Jang J, Shin N, Jung S, Kim B, et al. Prognostic value of computed tomography‐based volumetric body composition analysis in patients with head and neck cancer: Feasibility study. Head Neck. 2020;42:2614–25. 10.1002/hed.26310. 32543090 10.1002/hed.26310

[39] Erul E, Guven DC, Ozbay Y, Altunbulak AY, Kahvecioglu A, Ercan F, et al. Evaluation of sarcopenia as a prognostic biomarker in locally advanced head and neck squamous cell carcinoma. Biomark Med. 2023;17:87–99. 10.2217/bmm-2022-0748. 37042459 10.2217/bmm-2022-0748

[40] Findlay M, Brown C, De Abreu Lourenço R, White K, Bauer J. Sarcopenia and myosteatosis in patients undergoing curative radiotherapy for head and neck cancer: Impact on survival, treatment completion, hospital admission and cost. J Hum Nutr Diet. 2020;33:811–21. 10.1111/jhn.12788. 32609428 10.1111/jhn.12788

[41] Findlay M, White K, Brown C, Bauer JD. Nutritional status and skeletal muscle status in patients with head and neck cancer: impact on outcomes. J Cachexia Sarcopenia Muscle. 2021;12:2187–98. 10.1002/jcsm.12829. 34676673 10.1002/jcsm.12829 PMC8718020

[42] Galli A, Colombo M, Prizio C, Carrara G, Lira Luce F, Paesano PL, et al. Skeletal muscle depletion and major postoperative complications in locally-advanced head and neck cancer: a comparison between ultrasound of rectus femoris muscle and neck cross-sectional imaging. Cancers. 2022;14:347. 10.3390/cancers14020347. 35053512 10.3390/cancers14020347 PMC8774237

[43] Ganju RG, Morse R, Hoover A, TenNapel M, Lominska CE. The impact of sarcopenia on tolerance of radiation and outcome in patients with head and neck cancer receiving chemoradiation. Radiother Oncol. 2019;137:117–24. 10.1016/j.radonc.2019.04.023. 31085391 10.1016/j.radonc.2019.04.023

[44] Grossberg AJ, Chamchod S, Fuller CD, Mohamed ASR, Heukelom J, Eichelberger H, et al. Association of body composition with survival and locoregional control of radiotherapy-treated head and neck squamous cell carcinoma. JAMA Oncol. 2016;2:782. 10.1001/jamaoncol.2015.6339. 26891703 10.1001/jamaoncol.2015.6339 PMC5080910

[45] Haehl E, Alvino L, Rühle A, Zou J, Fabian A, Grosu AL, et al. Sarcopenia as a prognostic marker in elderly head and neck squamous cell carcinoma patients undergoing (chemo-)radiation. Cancers. 2022;14:5536. 10.3390/cancers14225536. 36428629 10.3390/cancers14225536 PMC9688610

[46] He WZ, Jiang C, Liu LL, Yin CX, Rong YM, Hu WM, et al. Association of body composition with survival and inflammatory responses in patients with non-metastatic nasopharyngeal cancer. Oral Oncol. 2020;108:104771. 10.1016/j.oraloncology.2020.104771. 32485608 10.1016/j.oraloncology.2020.104771

[47] Hua X, Li WZ, Huang X, Wen W, Huang HY, Long ZQ, et al. Modeling sarcopenia to predict survival for patients with nasopharyngeal carcinoma receiving concurrent chemoradiotherapy. Front Oncol. 2021;11:625534. 10.3389/fonc.2021.625534. 33777769 10.3389/fonc.2021.625534 PMC7993198

[48] Huang X, Ma J, Li L, Zhu X. Severe muscle loss during radical chemoradiotherapy for non‐metastatic nasopharyngeal carcinoma predicts poor survival. Cancer Med. 2019;8:6604–13. 10.1002/cam4.2538. 31517443 10.1002/cam4.2538 PMC6825977

[49] Huang X, Lv LN, Zhao Y, Li L, Zhu XD. Is skeletal muscle loss associated with chemoradiotherapy toxicity in nasopharyngeal carcinoma patients? A prospective study. Clin Nutr. 2021;40:295–302. 10.1016/j.clnu.2020.05.020. 32507513 10.1016/j.clnu.2020.05.020

[50] Huang CH, Lue KH, Chen PR, Hsieh TC, Chou YF. Association between sarcopenia and immediate complications and mortality in patients with oral cavity squamous cell carcinoma undergoing surgery. Cancers. 2022;14:785. 10.3390/cancers14030785. 35159050 10.3390/cancers14030785 PMC8833832

[51] Huiskamp LFJ, Chargi N, Devriese LA, De Jong PA, De Bree R. The predictive and prognostic value of low skeletal muscle mass for dose-limiting toxicity and survival in head and neck cancer patients receiving concomitant cetuximab and radiotherapy. Eur Arch Otorhinolaryngol. 2020;277:2847–58. 10.1007/s00405-020-05972-2. 32335709 10.1007/s00405-020-05972-2 PMC7496017

[52] Jin W, Rich B, Yechieli R, Freedman L, Samuels MA, Abramowitz M, et al. A single axial slice of the sternocleidomastoids and paravertebral muscles associated with worse local progression-free survival and severe toxicity in sarcopenic head and neck cancer patients undergoing radiotherapy. Cureus. 2022;14:e22463. 10.7759/cureus.22463. 35345685 10.7759/cureus.22463 PMC8942181

[53] Jones AJ, Campiti VJ, Alwani M, Novinger LJ, Tucker BJ, Bonetto A, et al. Sarcopenia is associated with blood transfusions in head and neck cancer free flap surgery. Laryngoscope Investig Otolaryngol. 2021;6:200–10. 10.1002/lio2.530. 33869752 10.1002/lio2.530 PMC8035950

[54] Jung AR, Roh JL, Kim JS, Choi SH, Nam SY, Kim SY. Efficacy of head and neck computed tomography for skeletal muscle mass estimation in patients with head and neck cancer. Oral Oncol. 2019;95:95–9. 10.1016/j.oraloncology.2019.06.009. 31345401 10.1016/j.oraloncology.2019.06.009

[55] Jung AR, Roh JL, Kim JS, Kim SB, Choi SH, Nam SY, et al. Prognostic value of body composition on recurrence and survival of advanced-stage head and neck cancer. Eur J Cancer. 2019;116:98–106. 10.1016/j.ejca.2019.05.006. 31185387 10.1016/j.ejca.2019.05.006

[56] Jung AhR, Roh JL, Kim JS, Choi SH, Nam SY, Kim SY. The impact of skeletal muscle depletion on older adult patients with head and neck cancer undergoing primary surgery. J Geriatr Oncol. 2021;12:128–33. 10.1016/j.jgo.2020.06.009. 32565144 10.1016/j.jgo.2020.06.009

[57] Karsten RT, Al‐Mamgani A, Bril SI, Tjon‐A‐Joe S, Van Der Molen L, De Boer JP, et al. Sarcopenia, a strong determinant for prolonged feeding tube dependency after chemoradiotherapy for head and neck cancer. Head Neck. 2019;41:4000–8. 10.1002/hed.25938. 31472000 10.1002/hed.25938

[58] Lee J, Liu S, Dai K, Huang Y, Li C, Chen JC, et al. Sarcopenia and systemic inflammation synergistically impact survival in oral cavity cancer. Laryngoscope. 2021;131:E1530–E1538. 10.1002/lary.29221. 33135827 10.1002/lary.29221

[59] Lee B, Bae YJ, Jeong WJ, Kim H, Choi BS, Kim JH. Temporalis muscle thickness as an indicator of sarcopenia predicts progression-free survival in head and neck squamous cell carcinoma. Sci Rep. 2021;11:19717. 10.1038/s41598-021-99201-3. 34611230 10.1038/s41598-021-99201-3 PMC8492642

[60] Lee J, Liu SH, Chen JCH, Leu YS, Liu CJ, Chen YJ. Progressive muscle loss is an independent predictor for survival in locally advanced oral cavity cancer: a longitudinal study. Radiother Oncol. 2021;158:83–9. 10.1016/j.radonc.2021.02.014. 33621588 10.1016/j.radonc.2021.02.014

[61] Lin S, Lin Y, Kang B, Yin C, Chang K, Chi C, et al. Sarcopenia results in poor survival rates in oral cavity cancer patients. Clin Otolaryngol. 2020;45:327–33. 10.1111/coa.13481. 31769607 10.1111/coa.13481

[62] Makiguchi T, Yamaguchi T, Nakamura H, Suzuki K, Harimoto N, Shirabe K, et al. Impact of skeletal muscle mass volume on surgical site infection in free flap reconstruction for oral cancer. Microsurgery. 2019;39:598–604. 10.1002/micr.30494. 31328303 10.1002/micr.30494

[63] Mascarella MA, Patel T, Vendra V, Gardiner L, Kergoat M, Kubik MW, et al. Poor treatment tolerance in head and neck cancer patients with low muscle mass. Head Neck. 2022;44:844–50. 10.1002/hed.26978. 35020252 10.1002/hed.26978 PMC11412609

[64] Mascarella MA, Gardiner L, Patel T, Vendra V, Khan N, Kergoat M, et al. Cervical paraspinal skeletal muscle index outperforms frailty indices to predict postoperative adverse events in operable head and neck cancer with microvascular reconstruction. Microsurgery. 2022;42:209–16. 10.1002/micr.30848. 34935198 10.1002/micr.30848

[65] McGoldrick DM, Yassin Alsabbagh A, Shaikh M, Pettit L, Bhatia SK. Masseter muscle defined sarcopenia and survival in head and neck cancer patients. Br J Oral Maxillofac Surg. 2022;60:454–8. 10.1016/j.bjoms.2021.07.020. 35339299 10.1016/j.bjoms.2021.07.020

[66] Morse R, Ganju RG, Gan GN, Cao Y, Neupane P, Kakarala K, et al. Sarcopenia and treatment toxicity in older adults undergoing chemoradiation for head and neck cancer: identifying factors to predict frailty. Int J Radiat Oncol. 2022;114:e295. 10.3390/cancers14092094. 10.3390/cancers14092094 PMC9103923 35565223

[67] Nagpal P, Pruthi DS, Pandey M, Yadav A, Singh H. Impact of sarcopenia in locally advanced head and neck cancer treated with chemoradiation: an Indian tertiary care hospital experience. Oral Oncol. 2021;121:105483. 10.1016/j.oraloncology.2021.105483. 34403887 10.1016/j.oraloncology.2021.105483

[68] Nishikawa D, Hanai N, Suzuki H, Koide Y, Beppu S, Hasegawa Y. The impact of skeletal muscle depletion on head and neck squamous cell carcinoma. ORL. 2018;80:1–9. 10.1159/000485515. 29393251 10.1159/000485515

[69] Pai PC, Chuang CC, Chuang WC, Tsang NM, Tseng CK, Chen KH, et al. Pretreatment subcutaneous adipose tissue predicts the outcomes of patients with head and neck cancer receiving definitive radiation and chemoradiation in Taiwan. Cancer Med. 2018;7:1630–41. 10.1002/cam4.1365. 29608254 10.1002/cam4.1365 PMC5943483

[70] Van Rijn-Dekker MI, Van Den Bosch L, Van Den Hoek JGM, Bijl HP, Van Aken ESM, Van Der Hoorn A, et al. Impact of sarcopenia on survival and late toxicity in head and neck cancer patients treated with radiotherapy. Radiother Oncol. 2020;147:103–10. 10.1016/j.radonc.2020.03.014. 32251949 10.1016/j.radonc.2020.03.014

[71] Shodo R, Yamazaki K, Ueki Y, Takahashi T, Horii A. Sarcopenia predicts a poor treatment outcome in patients with head and neck squamous cell carcinoma receiving concurrent chemoradiotherapy. Eur Arch Otorhinolaryngol. 2021;278:2001–9. 10.1007/s00405-020-06273-4. 32772234 10.1007/s00405-020-06273-4

[72] Stone L, Olson B, Mowery A, Krasnow S, Jiang A, Li R, et al. Association between sarcopenia and mortality in patients undergoing surgical excision of head and neck cancer. JAMA Otolaryngol Neck Surg. 2019;145:647. 10.1001/jamaoto.2019.1185. 10.1001/jamaoto.2019.1185 PMC6555480 31169874

[73] Takenaka Y, Takemoto N, Otsuka T, Nishio M, Tanida M, Fujii T, et al. Predictive significance of body composition indices in patients with head and neck squamous cell carcinoma treated with nivolumab: a multicenter retrospective study. Oral Oncol. 2022;132:106018. 10.1016/j.oraloncology.2022.106018. 35835055 10.1016/j.oraloncology.2022.106018

[74] Tamaki A, Manzoor NF, Babajanian E, Ascha M, Rezaee R, Zender CA. Clinical significance of sarcopenia among patients with advanced oropharyngeal cancer. Otolaryngol Neck Surg. 2019;160:480–7. 10.1177/0194599818793857. 10.1177/0194599818793857 30105922

[75] Thureau S, Lebret L, Lequesne J, Cabourg M, Dandoy S, Gouley C, et al. Prospective evaluation of sarcopenia in head and neck cancer patients treated with radiotherapy or radiochemotherapy. Cancers. 2021;13:753. 10.3390/cancers13040753. 33670339 10.3390/cancers13040753 PMC7917983

[76] Van Heusden HC, Chargi N, Dankbaar JW, Smid EJ, De Bree R. Masseter muscle parameters can function as an alternative for skeletal muscle mass assessments on cross-sectional imaging at lumbar or cervical vertebral levels. Quant Imaging Med Surg. 2022;12:15–27. 10.21037/qims-21-43. 34993057 10.21037/qims-21-43 PMC8666780

[77] Wendrich AW, Swartz JE, Bril SI, Wegner I, De Graeff A, Smid EJ, et al. Low skeletal muscle mass is a predictive factor for chemotherapy dose-limiting toxicity in patients with locally advanced head and neck cancer. Oral Oncol. 2017;71:26–33. 10.1016/j.oraloncology.2017.05.012. 28688687 10.1016/j.oraloncology.2017.05.012

[78] Willemsen ACH, De Moor N, Van Dessel J, Baijens LWJ, Bila M, Hauben E, et al. The predictive and prognostic value of weight loss and body composition prior to and during immune checkpoint inhibition in recurrent or metastatic head and neck cancer patients. Cancer Med. 2023;12:7699–712. 10.1002/cam4.5522. 36484469 10.1002/cam4.5522 PMC10134381

[79] Xing X, Zhou X, Yang Y, Li Y, Hu C, Shen C. The impact of body composition parameters on severe toxicities in patients with locoregionally advanced nasopharyngeal carcinoma undergoing neoadjuvant chemotherapy. Ann Transl Med. 2021;9:1180–1180. 10.21037/atm-21-3412. 34430621 10.21037/atm-21-3412 PMC8350723

[80] Yamahara K, Mizukoshi A, Lee K, Ikegami S. Sarcopenia with inflammation as a predictor of survival in patients with head and neck cancer. Auris Nasus Larynx. 2021;48:1013–22. 10.1016/j.anl.2021.03.021. 33883097 10.1016/j.anl.2021.03.021

[81] Yoshimura T, Suzuki H, Takayama H, Higashi S, Hirano Y, Tezuka M, et al. Impact of preoperative low prognostic nutritional index and high intramuscular adipose tissue content on outcomes of patients with oral squamous cell carcinoma. Cancers. 2020;12:3167. 10.3390/cancers12113167. 33126582 10.3390/cancers12113167 PMC7692578

[82] Yunaiyama D, Okubo M, Arizono E, Tsukahara K, Tanigawa M, Nagao T, et al. Sarcopenia at the infrahyoid level as a prognostic factor in patients with advanced-stage non-virus-related head and neck carcinoma. Eur Arch Otorhinolaryngol. 2022;279:3131–7. 10.1007/s00405-021-07147-z. 34697649 10.1007/s00405-021-07147-z

[83] Swartz JE, Pothen AJ, Wegner I, Smid EJ, Swart KMA, De Bree R, et al. Feasibility of using head and neck CT imaging to assess skeletal muscle mass in head and neck cancer patients. Oral Oncol. 2016;62:28–33. 10.1016/j.oraloncology.2016.09.006. 27865369 10.1016/j.oraloncology.2016.09.006

[84] Morley JE, Baumgartner RN, Roubenoff R, Mayer J, Nair KS. Sarcopenia. J Lab Clin Med. 2001;137:231–43. 10.1067/mlc.2001.113504. 11283518 10.1067/mlc.2001.113504

[85] Nelke C, Dziewas R, Minnerup J, Meuth SG, Ruck T. Skeletal muscle as potential central link between sarcopenia and immune senescence. eBioMedicine. 2019;49:381–8. 10.1016/j.ebiom.2019.10.034. 31662290 10.1016/j.ebiom.2019.10.034 PMC6945275

[86] Cole CL, Kleckner IR, Jatoi A, Schwarz EM, Dunne RF. The role of systemic inflammation in cancer‐associated muscle wasting and rationale for exercise as a therapeutic intervention. JCSM Clin Rep. 2018;3:1–19. PMC6534125 31134216

[87] Bosaeus I. Nutritional support in multimodal therapy for cancer cachexia. Support Care Cancer. 2008;16:447–51. 10.1007/s00520-007-0388-7. 18196284 10.1007/s00520-007-0388-7

[88] Capitão C, Coutinho D, Neves PM, Capelas ML, Pimenta NM, Santos T, et al. Protein intake and muscle mass maintenance in patients with cancer types with high prevalence of sarcopenia: a systematic review. Support Care Cancer. 2022;30:3007–15. 10.1007/s00520-021-06633-8. 34697674 10.1007/s00520-021-06633-8

[89] the PreMiO Study Group, Muscaritoli M, Lucia S, Farcomeni A, Lorusso V, Saracino V, et al. Prevalence of malnutrition in patients at first medical oncology visit: the PreMiO study. Oncotarget. 2017;8:79884–96. 10.18632/oncotarget.20168. 29108370 10.18632/oncotarget.20168 PMC5668103

[90] Petermann‐Rocha F, Balntzi V, Gray SR, Lara J, Ho FK, Pell JP, et al. Global prevalence of sarcopenia and severe sarcopenia: a systematic review and meta‐analysis. J Cachexia Sarcopenia Muscle. 2022;13:86–99. 10.1002/jcsm.12783. 34816624 10.1002/jcsm.12783 PMC8818604

[91] Shachar SS, Williams GR, Muss HB, Nishijima TF. Prognostic value of sarcopenia in adults with solid tumours: a meta-analysis and systematic review. Eur J Cancer. 2016;57:58–67. 10.1016/j.ejca.2015.12.030. 26882087 10.1016/j.ejca.2015.12.030

[92] Rier HN, Jager A, Sleijfer S, Maier AB, Levin MD. The prevalence and prognostic value of low muscle mass in cancer patients: a review of the literature. Oncologist. 2016;21:1396–409. 10.1634/theoncologist.2016-0066. 27412391 10.1634/theoncologist.2016-0066 PMC5189631

[93] Buckinx F, Landi F, Cesari M, Fielding RA, Visser M, Engelke K, et al. Pitfalls in the measurement of muscle mass: a need for a reference standard. J Cachexia Sarcopenia Muscle. 2018;9:269–78. 10.1002/jcsm.12268. 29349935 10.1002/jcsm.12268 PMC5879987

[94] Van Heusden HC, Swartz JE, Chargi N, De Jong PA, Van Baal MCPM, Wegner I, et al. Feasibility of assessment of skeletal muscle mass on a single cross-sectional image at the level of the fourth thoracic vertebra. Eur J Radio. 2021;142:109879. 10.1016/j.ejrad.2021.109879. 10.1016/j.ejrad.2021.109879 34343845

[95] Van Vugt JLA, Levolger S, Gharbharan A, Koek M, Niessen WJ, Burger JWA, et al. A comparative study of software programmes for cross‐sectional skeletal muscle and adipose tissue measurements on abdominal computed tomography scans of rectal cancer patients. J Cachexia Sarcopenia Muscle. 2017;8:285–97. 10.1002/jcsm.12158. 27897414 10.1002/jcsm.12158 PMC5697014

[96] Zwart AT, Cavalheiro VJ, Lamers MJ, Dierckx RAJO, De Bock GH, Halmos GB, et al. The validation of low-dose CT scans from the [18F]-FDG PET-CT scan to assess skeletal muscle mass in comparison with diagnostic neck CT scans. Eur J Nucl Med Mol Imaging. 2023;50:1735–42. 10.1007/s00330-020-07440-1. 36781423 10.1007/s00259-023-06117-3 PMC10119057

[97] Zwart AT, Becker JN, Lamers MJ, Dierckx RAJO, De Bock GH, Halmos GB, et al. Skeletal muscle mass and sarcopenia can be determined with 1.5-T and 3-T neck MRI scans, in the event that no neck CT scan is performed. Eur Radio. 2021;31:4053–62. 10.1007/s00259-023-06117-3. 10.1007/s00330-020-07440-1 PMC8128750 33219847

[98] Prado CM, Lieffers JR, McCargar LJ, Reiman T, Sawyer MB, Martin L, et al. Prevalence and clinical implications of sarcopenic obesity in patients with solid tumours of the respiratory and gastrointestinal tracts: a population-based study. Lancet Oncol. 2008;9:629–35. 10.1016/s1470-2045(08)70153-0. 18539529 10.1016/S1470-2045(08)70153-0

[99] Martin L, Birdsell L, MacDonald N, Reiman T, Clandinin MT, McCargar LJ, et al. Cancer cachexia in the age of obesity: skeletal muscle depletion is a powerful prognostic factor, independent of body mass index. J Clin Oncol. 2013;31:1539–47. 10.1200/jco.2012.45.2722. 23530101 10.1200/JCO.2012.45.2722

[100] Ye Z, Saraf A, Ravipati Y, Hoebers F, Catalano PJ, Zha Y, et al. Development and validation of an automated image-based deep learning platform for sarcopenia assessment in head and neck cancer. JAMA Netw Open. 2023;6:e2328280. 10.1001/jamanetworkopen.2023.28280. 37561460 10.1001/jamanetworkopen.2023.28280 PMC10415962

[101] Naser MA, Wahid KA, Grossberg AJ, Olson B, Jain R, El-Habashy D, et al. Deep learning auto-segmentation of cervical skeletal muscle for sarcopenia analysis in patients with head and neck cancer. Front Oncol. 2022;12:930432. 10.3389/fonc.2022.930432. 35965493 10.3389/fonc.2022.930432 PMC9366009

[102] Hua X, Liu S, Liao JF, Wen W, Long ZQ, Lu ZJ, et al. When the loss costs too much: a systematic review and meta-analysis of sarcopenia in head and neck cancer. Front Oncol. 2020;9:1561. 10.3389/fonc.2019.01561. 32117787 10.3389/fonc.2019.01561 PMC7012991

[103] Findlay M, White K, Lai M, Luo D, Bauer JD. The association between computed tomography–defined sarcopenia and outcomes in adult patients undergoing radiotherapy of curative intent for head and neck cancer: a systematic review. J Acad Nutr Diet. 2020;120:1330–.e8. 10.1016/j.jand.2020.03.021. 32711854 10.1016/j.jand.2020.03.021

[104] Findlay M, White K, Stapleton N, Bauer J. Is sarcopenia a predictor of prognosis for patients undergoing radiotherapy for head and neck cancer? A meta-analysis. Clin Nutr. 2021;40:1711–8. 10.1016/j.clnu.2020.09.017. 32994071 10.1016/j.clnu.2020.09.017

[105] Takenaka Y, Takemoto N, Oya R, Inohara H. Prognostic impact of sarcopenia in patients with head and neck cancer treated with surgery or radiation: a meta-analysis. PLOS ONE. 2021;16:e0259288. 10.1371/journal.pone.0259288. Birkeland A, editor 34714876 10.1371/journal.pone.0259288 PMC8555817

[106] Wong A, Zhu D, Kraus D, Tham T. Radiologically defined sarcopenia affects survival in head and neck cancer: a meta‐analysis. Laryngoscope. 2021;131:333–41. 10.1002/lary.28616. 32220072 10.1002/lary.28616

[107] Surov A, Wienke A. Low skeletal muscle mass predicts relevant clinical outcomes in head and neck squamous cell carcinoma. A meta analysis. Ther Adv Med Oncol. 2021;13:175883592110088. 10.1177/17588359211008844. 10.1177/17588359211008844 PMC8127787 34035838

[108] Edwards A, Hughes BGM, Brown T, Bauer J. Prevalence and impact of computed tomography–defined sarcopenia on survival in patients with human papillomavirus–positive oropharyngeal cancer: a systematic review. Adv Nutr. 2022;13:2433–44. 10.1093/advances/nmac076. 35876662 10.1093/advances/nmac076 PMC9776633

[109] Graves JP, Daher GS, Bauman MMJ, Moore EJ, Tasche KK, Price DL, et al. Association of sarcopenia with oncologic outcomes of primary treatment among patients with oral cavity cancer: a systematic review and meta-analysis. Oral Oncol. 2023;147:106608. 10.1016/j.oraloncology.2023.106608. 37897858 10.1016/j.oraloncology.2023.106608

