# Supplementary material for: The predictive and prognostic role of radiologically defined sarcopenia in head and neck cancer: a systematic review and multi-level meta-analysis
Source: Br J Cancer. 2025 Jun 2;133(2):131–43. doi: 10.1038/s41416-025-03049-7 (PMC12304306; doi:10.1038/s41416-025-03049-7)
Supplement: Supplementary file 2 — search strategy [file 41416_2025_3049_MOESM2_ESM.docx]

**Supplementary data S2. Search strategy**

**Pubmed:**

(("Magnetic Resonance Imaging"[Mesh] OR ("magnetic resonance"[tiab] AND (image[tiab] OR images[tiab] OR imaging[tiab])) OR mri[tiab] OR mris[tiab] OR nmr[tiab] OR mra[tiab] OR mras[tiab] OR zeugmatograph*[tiab] OR "mr tomography"[tiab] OR "mr tomographies"[tiab] OR "mr tomographic"[tiab] OR "proton spin"[tiab] OR ((magneti*[tiab] OR "chemical shift"[tiab]) AND imaging[tiab]) OR fmri[tiab] OR fmri[tiab] OR "Tomography, X-Ray Computed"[Mesh] OR computed tomograph*[tiab] OR ct[tiab] OR cts[tiab] OR cat scan*[tiab] OR computer assisted tomograph*[tiab] OR computerized tomograph*[tiab] OR computerized tomograph*[tiab] OR computed x ray tomograph*[tiab] OR computed xray tomograph*[tiab] )

 AND

(body composition[Title/Abstract] OR sarcopen*[Title/Abstract] OR cachexi*[Title/Abstract] OR malnutrition*[Title/Abstract] OR muscle mass[Title/Abstract] OR muscular mass[Title/Abstract] OR muscle loss[Title/Abstract] OR muscular loss[Title/Abstract]))

**Embase**:

('body composition'/de OR 'body composition' OR (body:ab,ti,kw AND composition:ab,ti,kw) OR ‘sarcopenia’/exp OR (sarcopenia:ab,ti,kw) OR ‘cachexia’/exp OR (cachexia:ab,ti,kw) OR ‘muscle mass’/exp OR (muscle:ab,ti,kw AND mass:ab,ti,kw) OR ‘muscle loss’/exp OR (muscle:ti,ab,kw AND loss:ti,ab,kw))

AND

('nuclear magnetic resonance imaging'/exp) OR MRI:ab,ti,kw OR magnetic resonance imaging:ab,ti,kw OR 'computer assisted tomography'/exp OR (CT:ab,ti,kw or CT-imaging:ab,ti,kw)
